# Lifestyle‐Related Risk Factors for Pancreatic Ductal Adenocarcinoma: A Longitudinal Analysis of 1,120,377 Individuals From the NHISS Cohort

**DOI:** 10.1002/cam4.70848

**Published:** 2025-04-06

**Authors:** Hyunseok Jee

**Affiliations:** ^1^ School of Kinesiology Yeungnam University Gyeongsan Gyeongbuk South Korea

**Keywords:** exercise, exercise program development, pancreatic cancer, receiver operating characteristic curve analysis, risk factors

## Abstract

**Objective:**

Utilizing data from the National Health Insurance Sharing Service database, this study explored significant risk factors for pancreatic cancer in a cohort of 1,120,377 South Korean individuals over a 10‐year period (2009–2019).

**Methods:**

Propensity score matching was employed to ensure comparability between 3535 pancreatic cancer patients and a control group with a common cold diagnosis. The study analyzed various lifestyle factors and biochemical markers, including smoking status, alcohol consumption, fasting blood glucose (FBS) levels, liver enzyme levels, and Charlson comorbidity index (CCI) scores.

**Results:**

The findings revealed that current smoking, frequent alcohol consumption, and elevated levels of FBS and liver enzymes were associated with an increased risk of pancreatic cancer. Conversely, engaging in high‐intensity exercise (≥ 20 min, twice weekly) was correlated with a 20% reduction in pancreatic cancer risk (*p* < 0.05). Additionally, optimal thresholds for total cholesterol (179.50 mg/dL), GGT (29.50 U/L), low‐density lipoprotein cholesterol (104.50 mg/dL), and CCI score (2.50) were identified, which may facilitate early diagnosis and intervention.

**Conclusions:**

These findings underscore the importance of modifiable lifestyle factors in managing pancreatic cancer risk and highlight the potential of personalized, evidence‐based interventions—such as high‐intensity exercise programs—in improving prevention and treatment outcomes.

**Abbreviations**
AUC: area under the curve
BMI: body mass index
CCI: Charlson comorbidity index
CI: confidence intervals
DB: database
FBS: fasting blood glucose
GGT: gamma‐glutamyl transferase
KSCDCR: Korean Standard Classification of Disease and Causes of Death
LDL: low‐density lipoprotein cholesterol
NHISS: National Health Insurance Sharing Service
OR: odds ratio
PSM: propensity score matching
ROC: receiver operating characteristic
SCr: serum creatinine
SGOT: serum glutamic oxaloacetic transaminase
SGPT: serum glutamic pyruvic transaminase
SBP: systolic blood pressure
TC: total cholesterol

## 1 Introduction

Pancreatic cancer is a malignant tumor, with over 90% of cases classified as ductal adenocarcinoma [1, 2]. Although less common compared to some other cancers, pancreatic cancer is extremely lethal, often metastasizing by the time of its diagnosis, and is associated with a high mortality rate. The incidence of this cancer is notably higher in high‐income countries. It occurs predominantly in older adults, with a higher prevalence among men typically aged between 60 and 80 years compared to women in the same age group [3, 4, 5]. The five‐year survival rate of pancreatic cancer is below 10%, primarily owing to difficulties associated with its early detection, which often result in late‐stage diagnosis [6]. Several factors contribute to the development of pancreatic cancer, including family history and genetic mutations. Research indicates that mutations in tumor suppressor genes, such as BRCA2, can substantially elevate the risk of developing the disease [7].

Environmental factors also play a critical role, with smoking identified as a major risk factor. Furthermore, obesity, diabetes, chronic pancreatitis, and alcohol abuse have been linked to an increased risk of pancreatic cancer [8, 9]. Dietary factors, such as a high‐calorie intake from processed meats and high‐fat foods, are also believed to increase cancer risk.

Amid ongoing research into personalized treatments based on genetic mutations, emerging therapies, such as immunotherapy, oncolytic virus therapy, and targeted therapies, are demonstrating limited but increasing success [10, 11, 12, 13]. Recently, physical activities have been associated with a reduced risk of pancreatic cancer. Studies have reported that daily high‐intensity exercise for 20 min could lower pancreatic cancer risk by 50%, while 30 min of daily moderate‐intensity exercise could reduce the risk by 46% among men (*p* < 0.05) [14]. While the benefits of exercise for cancers, such as breast, prostate, and colorectal cancer—such as reduced recurrence risk, improved survival rates, reduced fatigue, and alleviated depression—have been widely studied, research on pancreatic cancer in this context remains limited. However, recent studies have explored the feasibility and impact of exercise interventions in pancreatic cancer patients. A randomized controlled trial demonstrated that progressive resistance training can improve physical fitness and body weight in pancreatic cancer patients [15]. Additionally, combined exercise programs have been assessed for their feasibility in patients with advanced pancreatic or lung cancer, showing potential benefits for overall well‐being [16]. A scoping review further supports the role of exercise in managing pancreatic cancer during treatment, highlighting its positive impact on patient outcomes [17]. To bridge this gap, the current study analyzed data from a 1‐million‐person cohort in the National Health Insurance Sharing Service (NHISS) database (DB), aiming to offer insights into the positive impacts of various types of exercise on pancreatic cancer risk and overall health outcomes. The primary focus of this study was to evaluate modifiable lifestyle risk factors associated with pancreatic ductal adenocarcinoma (PDAC), the most common and aggressive form of pancreatic cancer. While genetic predispositions and tumor‐specific factors, such as PDAC stage, are crucial in understanding disease progression, this study prioritized modifiable lifestyle factors due to their potential for early intervention and prevention. The NHISS dataset, which does not include genetic data or detailed tumor staging information, allowed for a large‐scale epidemiological assessment of lifestyle behaviors in relation to PDAC risk. Our selection of key risk factors—including smoking, alcohol consumption, metabolic indicators (fasting blood glucose and liver enzymes), and exercise—was based on prior literature identifying their strong associations with pancreatic carcinogenesis. While obesity and chronic pancreatitis are known risk factors, specific data on chronic pancreatitis were not available in the NHISS dataset. Instead, we included body mass index (BMI) and liver enzyme levels (gamma‐glutamyl transferase [GGT], serum glutamic pyruvic transaminase [SGPT]) as indirect markers of metabolic dysfunction, which are known to influence PDAC risk.

Overall, this study focused on the latest data (2024) from the NHISS DB to analyze specific factors influencing pancreatic cancer incidence from multiple perspectives. Specifically, using data collected from 1,120,377 individuals, this study aimed to identify modifiable lifestyle factors and metabolic indicators that may contribute to pancreatic cancer development, with a focus on their potential role in early prevention strategies.

## 2 Methods

### 2.1 Study Design

This longitudinal study used data from the NHISS DB, which includes comprehensive medical records of the South Korean population over a 10‐year period (2009–2019). These records represent the most recent NHISS data available as of 2024. Pancreatic‐cancer‐related diagnostic codes were sourced from the Korean Standard Classification of Disease and Causes of Death (KSCDCR) to identify patients with pancreatic cancer (Table S1). These codes include C25 (malignant neoplasm of the pancreas) and its subcategories, such as C25.0 (malignant neoplasm of the head of the pancreas), C25.1 (malignant neoplasm of the body of the pancreas), C25.2 (malignant neoplasm of the tail of the pancreas), C25.3 (malignant neoplasm of the pancreatic duct), C25.4 (malignant neoplasm of the endocrine pancreas), C25.7 (malignant neoplasm of other parts of the pancreas), C25.8 (malignant neoplasm of overlapping lesions of the pancreas), and C25.9 (malignant neoplasm of the pancreas, unspecified).

Notably, all hospitals in South Korea are required to submit medical records to the Health Insurance Review and Assessment Service [18]. Furthermore, the government documents all cancer cases, including those of pancreatic cancer, and reports them to the World Health Organization. Physicians initially diagnose pancreatic cancer based on clinical suspicion and subsequently confirm it through clinical or histological assessments, including biopsies or computed tomography scans. Follow‐up records, including data on recurrences, metastases, or new cancers, are also documented.

**FIGURE 1:**
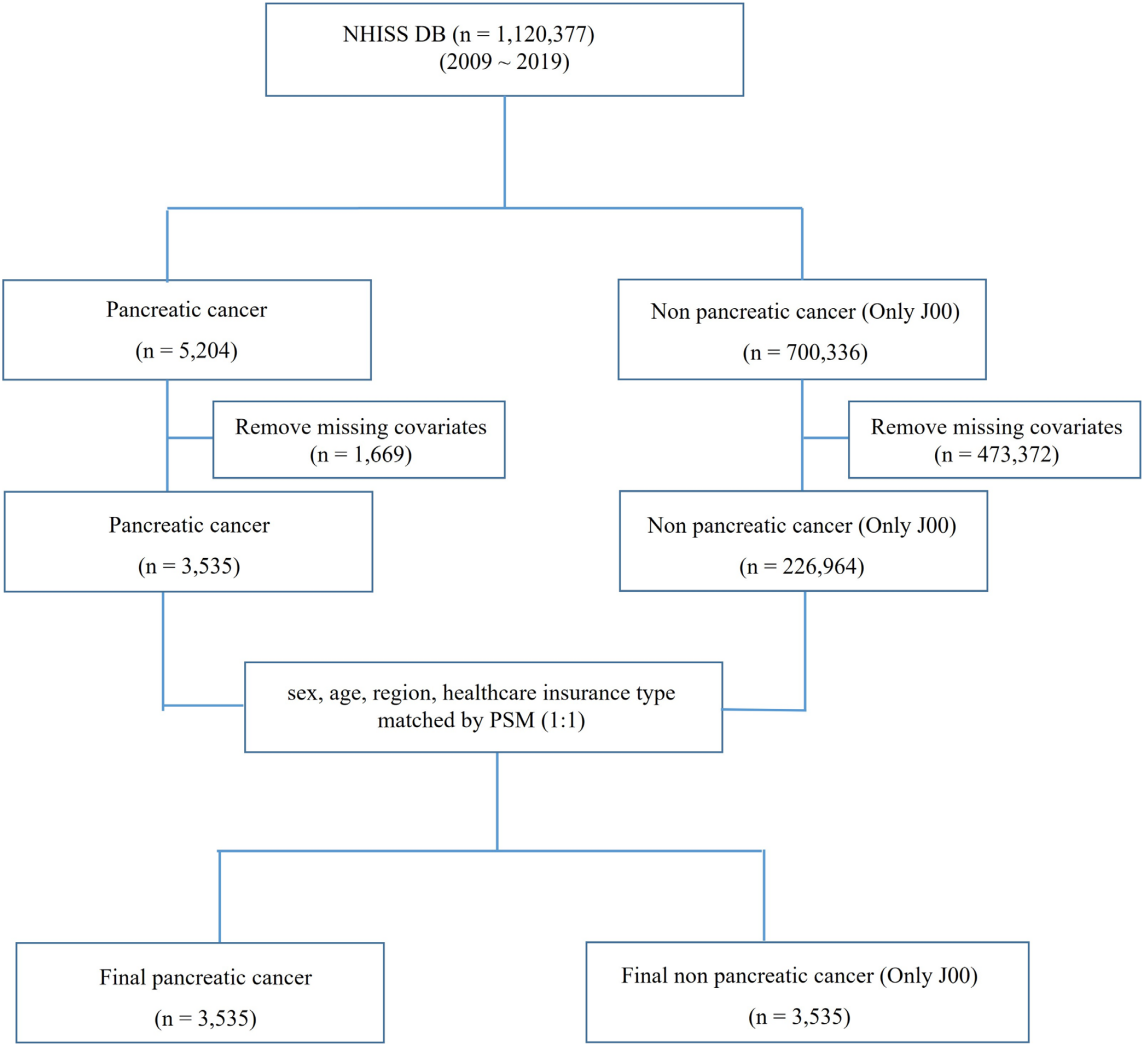
Study flowchart detailing the data origin and analysis parameters for pancreatic cancer patients. NHISS data were used in this study. Out of the 1,120,377 registered patients, the records of 5204 pancreatic cancer patients and 700,336 common cold patients were extracted. After eliminating missing covariates, 1:1 PSM was performed based on sex, age, region, and type of healthcare insurance. Ultimately, 3535 pancreatic cancer patients and 3535 common cold patients were selected for the analysis. The 21 parameters under evaluation were subjected to *t*‐test, logistic regression, and ROC curve analyses. NHISS DB, National Health Insurance Sharing Service database; PSM, propensity score matching; ROC, Receiver operating characteristic.

To identify key factors associated with pancreatic cancer, we compared patients diagnosed with pancreatic cancer to a control group, analyzing 21 variables (Figure 1). The control group comprised of patients diagnosed with the common cold (coded J00), selected as a low‐severity condition to represent the healthiest individuals in the NHISS DB (Table 2).

Variables related to carcinogenesis in pancreatic organs were sourced from the NHISS DB. All variables analyzed in this study were collected prior to the diagnosis of pancreatic cancer, as part of routine national health checkups recorded in the NHISS DB. Cancer diagnoses, including pancreatic cancer, were subsequently identified through follow‐up records. This design ensured that lifestyle behaviors and metabolic measurements were assessed before cancer onset, allowing their evaluation as potential risk factors.

The study identified optimal threshold cutoff points for these significant variables using logistic regression analysis. This approach is crucial for developing factor‐based programs to predict pancreatic cancer progression and improve symptom management.

### 2.2 Data Sources and Subject Population

In South Korea, all citizens are required to undergo routine medical checkups and register their health status, enabling comprehensive tracking of their medical histories. The NHISS maintains comprehensive digital records of each patient's medical history from 2002 onwards [19]. NHISS data samples, drawn from a national cohort of health surveys and hospital prescription data, are accessible for research purposes. The dataset for this study was finalized after a comprehensive survey of the public NHISS data and the application of specific inclusion and exclusion criteria to a cohort of one million individuals.

Notably, the NHISS DB does not focus on particular diseases but collects data from individuals who undergo medical checkups, providing a representative snapshot of the population regardless of their disease status. This research DB includes the medical records of 1,120,377 individuals, representing approximately 2% of the total South Korean population of 50 million. These individuals are randomly selected to represent the broader demographic and can be grouped by variables, such as sex, age, medical insurance premiums, regional location, and other demographic features.

### 2.3 Variables Used in This Study

As stated previously, this study, conducted in 2024, analyzed the most recent data from the NHISS DB, which includes records from 2009 to 2019. The 21 variables examined were tracked from 2009 to 2019. Data for these variables were initially recorded from 2009 to 2015, with further revisions made from 2016 to 2019. The specific variables used in the analysis included the following:

Alcohol consumption data were recorded as the “number of drinks per week” between 2009 and 2015. From 2016 to 2019, these data were revised to reflect the “number of drinks in the past year.” Smoking status was categorized into three groups: (1) never smoked, (2) former smoker, and (3) current smoker. Additional variables included the following: height (cm), weight (kg), waist circumference (cm), BMI (kg/m^2^), systolic blood pressure (SBP, mmHg), diastolic blood pressure (mmHg), urinary protein levels (graded from 1 = weakly positive, 2 = positive [+1], 3 = positive [+2], 4 = positive [+3], to 5 = positive [+4]), hemoglobin (g/dL), fasting blood glucose (mg/dL), total cholesterol (TC, mg/dL), triglycerides (mg/dL), high‐density lipoprotein cholesterol (mg/dL), low‐density lipoprotein cholesterol (LDL, mg/dL), serum creatinine (SCr, mg/dL), serum glutamic oxaloacetic transaminase (SGOT)/aspartate aminotransferase (U/L), SGPT/alanine aminotransaminase (ALT) (U/L), and GGT (U/L).

For physical activity data, between 2009 and 2015, respondents reported the number of times they engaged in “20 min or more of vigorous exercise per week” or “30 min or more of moderate exercise per week.” From 2016 to 2019, this question was revised, and respondents reported the frequency of their participation in “vigorous physical activity in a week” or “moderate physical activity in a week.”

The 21 variables included in this study were selected based on previous research identifying their strong associations with cancer development, particularly in relation to lifestyle, metabolic dysfunction, and comorbidities. These variables were also chosen because they are routinely collected and standardized within the NHISS health examination framework, ensuring data completeness and comparability across the large cohort.

This study was exempt from ethical approval by the Institutional Review Board of Yeungnam University (IRB #7002016‐E‐2022‐015) because all participant data were anonymized. Identification was limited to numerical IDs to ensure confidentiality.

### 2.4 Statistical Analyses

The statistical analyses conducted in this study included chi‐square tests and paired *t*‐tests to assess differences among groups. Furthermore, logistic regression was used to evaluate the associations between the 21 study variables and pancreatic carcinogenesis. For significant factors, odds ratios (ORs), *p* values, and 95% confidence intervals (CI) were calculated. Moreover, a receiver operating characteristic (ROC) curve analysis was conducted to determine the optimal cutoff points for these significant variables.

All statistical analyses were performed using SAS software version 9.4 (SAS Institute, Cary, NC, USA) and R software version 4.3.1, considering a *p* value of less than 0.05 as statistically significant [20]. All data reported in this paper are presented as mean ± standard deviation.

## 3 Results

### 3.1 Data Characteristics

The NHISS DB, containing 10‐year medical records of 1,120,377 South Koreans, enables detailed tracking of disabilities, diseases, and medical treatments (Table 1). In this study, pancreatic cancer and nonpancreatic cancer groups were matched using propensity score matching (PSM) based on gender, age, region, and insurance status. This process resulted in 3535 individuals in each group (Figure 1).

**TABLE 1:** Characteristics of the NHISS‐DB‐based study population.

Variable	All		Pancreatic cancer		Nonpancreatic cancer		*p*
	(*n* = 7070)		(*n* = 3535)		(*n* = 3535)		
	*N* or mean	% or SD	*N* or mean	% or SD	*N* or mean	% or SD	
Ht	160.40	9.30	160.69	9.28	160.11	9.32	0.009
Wt	62.03	11.17	62.27	11.53	61.78	10.79	0.065
WC	82.42	8.90	82.53	9.17	82.32	8.63	0.314
BMI	24.03	3.28	24.03	3.44	24.02	3.12	0.834
SBP	126.23	15.60	126.13	15.46	126.33	15.74	0.573
DBP	77.07	9.94	76.93	9.89	77.20	9.98	0.253
Hb	13.70	1.60	13.72	1.63	13.68	1.57	0.428
FBS	107.21	33.44	111.63	38.39	102.79	26.92	< 0.0001
TC	193.18	39.95	189.60	40.48	196.75	39.09	< 0.0001
TG	136.45	101.18	136.97	111.47	135.93	89.74	0.666
HDL	54.62	22.15	54.11	18.90	55.13	24.97	0.053
LDL	113.00	45.85	109.09	36.56	116.91	53.27	< 0.0001
SCr	0.95	0.78	0.93	0.70	0.98	0.85	0.010
SGOT	28.16	22.48	29.65	27.50	26.67	15.82	< 0.0001
SGPT	25.86	24.01	27.58	29.45	24.14	16.73	< 0.0001
GGT	43.71	77.14	50.40	94.93	37.03	52.91	< 0.0001
*Urine protein*							
Negative (−)	6564	92.84	3253	92.02	3311	93.66	0.070
Weakly positive (±)	214	3.03	118	3.34	96	2.72	
Positive (+1)	173	2.45	92	2.60	81	2.29	
Positive (+2)	81	1.15	52	1.47	29	0.82	
Positive (+3)	31	0.44	16	0.45	15	0.42	
Positive (+4)	7	0.10	4	0.11	3	0.08	
*Smoking status*							
Never	4613	65.25	2249	63.62	2364	66.87	0.0002
Former	1272	17.99	630	17.82	642	18.16	
Current	1185	16.76	656	18.56	529	14.96	
*Alcohol consumption (1 week)*							
Average	0.89	1.62	0.92	1.66	0.86	1.57	0.081
0	4603	65.11	2291	64.81	2312	65.40	0.020
1	947	13.39	463	13.10	484	13.69	
2	563	7.96	260	7.36	303	8.57	
3	437	6.18	246	6.96	191	5.40	
4	130	1.84	69	1.95	61	1.73	
5	135	1.91	72	2.04	63	1.78	
6	79	1.12	34	0.96	45	1.27	
7	176	2.49	100	2.83	76	2.15	
*High‐intensity exercise (1 week)*							
Average	1.00	1.77	0.99	1.77	1.00	1.77	0.316
0	4659	65.90	2356	66.65	2303	65.15	0.007
1	695	9.83	327	9.25	368	10.41	
2	552	7.81	248	7.02	304	8.60	
3	456	6.45	238	6.73	218	6.17	
4	181	2.56	93	2.63	88	2.49	
5	216	3.06	129	3.65	87	2.46	
6	102	1.44	47	1.33	55	1.56	
7	209	2.96	97	2.74	112	3.17	
*Moderate‐intensity exercise (1 week)*							
Average	1.33	2.02	1.36	2.04	1.31	2.00	0.915
0	4115	58.20	2064	58.39	2051	58.02	0.044
1	708	10.01	330	9.34	378	10.69	
2	620	8.77	289	8.18	331	9.36	
3	603	8.53	315	8.91	288	8.15	
4	274	3.88	148	4.19	126	3.56	
5	274	3.88	155	4.38	119	3.37	
6	145	2.05	68	1.92	77	2.18	
7	331	4.68	166	4.70	165	4.67	
*CCI comorbidities*							
Acute myocardial infarction	139	1.97	82	2.32	57	1.61	0.032
Congestive heart failure	324	4.58	197	5.57	127	3.59	< 0.0001
Peripheral vascular accident	1111	15.71	590	16.69	521	14.74	0.024
Cerebrovascular accident	1036	14.65	557	15.76	479	13.55	0.009
Dementia	0	0.00	0	0.00	0	0.00	—
Pulmonary disease	3061	43.30	1705	48.23	1356	38.36	< 0.0001
Connective tissue disorder	431	6.10	271	7.67	160	4.53	< 0.0001
Peptic ulcer	3062	43.31	1737	49.14	1325	37.48	< 0.0001
Liver disease	540	7.64	388	10.98	152	4.30	< 0.0001
Diabetes	2221	31.41	1441	40.76	780	22.07	< 0.0001
Diabetes complications	644	9.11	438	12.39	206	5.83	< 0.0001
Paraplegia	56	0.79	25	0.71	31	0.88	0.421
Renal disease	246	3.48	161	4.55	85	2.40	< 0.0001
Cancer	862	12.19	599	16.94	263	7.44	< 0.0001
Metastatic cancer	133	1.88	109	3.08	24	0.68	< 0.0001
Severe liver disease	54	0.76	30	0.85	24	0.68	0.412
HIV	0	0.00	0	0.00	0	0.00	—
CCI score	2.28	2.08	2.78	2.26	1.77	1.74	< 0.0001
*CCI category*							
CCI score = 0	1383	19.56	459	12.98	924	26.14	< 0.0001
1 ≤ CCI score < 3	3136	44.36	1455	41.16	1681	47.55	
3 ≤ CCI score	2551	36.08	1621	45.86	930	26.31	

*Note:* Demographic characteristics of the study population derived from the NHISS DB. Data are presented as numbers, means, percentages, or standard deviations (SD).

Abbreviations: BMI, body mass index (kg/m^2^); CCI, Charlson comorbidity index; DBP, diastolic blood pressure (mmHg); FBS, fasting blood sugar (mg/dL); GGT, gamma‐glutamyl transferase (U/L); Hb, hemoglobin (g/dL); HDL, high‐density lipoprotein cholesterol (mg/dL); Ht, height (cm); LDL, low‐density lipoprotein cholesterol (mg/dL); NHISS DB, National Health Insurance Sharing Service database; SBP, systolic blood pressure (mmHg); SCr, serum creatinine (mg/dL); SGOT, serum glutamic oxaloacetic transaminase/aspartate aminotransferase (U/L); SGPT, serum glutamic pyruvic transaminase/alanine aminotransaminase (U/L); TC, total cholesterol (mg/dL); TG, glycerides (mg/dL); urine protein, protein in urine; WC, waist circumference (cm); Wt, weight (kg).

Subsequently, data for the 3535 pancreatic cancer patients were extracted from the NHISS DB using pancreatic cancer diagnostic codes provided by the KSCDCR, as outlined in Section 2. A control group comprising 3535 individuals with the common cold was also included for comparison.

Demographic analysis revealed that fasting blood sugar (FBS), SGOT, SGPT, and GGT levels were significantly higher in the pancreatic cancer group (*p* < 0.0001, Table 1) compared to the nonpancreatic cancer group. In contrast, higher TC, LDL, and SCr levels were more common in the nonpancreatic cancer group (*p* < 0.05). Smoking status also displayed significant differences (*p* = 0.0002). A greater proportion of individuals who had never smoked were included in the nonpancreatic cancer group (2249 ± 63.62 vs. 2364 ± 66.87). A similar trend was observed for those who had quit smoking (630 ± 17.82 vs. 642 ± 18.16). Conversely, the majority of current smokers belonged to the pancreatic cancer group (656 ± 18.56 vs. 529 ± 14.96).

Furthermore, alcohol consumption varied significantly. In particular, individuals who reported consuming alcohol three to five times per week showed a significantly higher incidence of pancreatic cancer compared to those with lower alcohol consumption frequencies (*p* = 0.020, Table 1). In terms of exercise, individuals who engaged in 20 min or more of vigorous activity on 1, 2, 6, or all 7 days per week were more common in the nonpancreatic cancer group. Conversely, individuals who engaged in vigorous exercise three to five times per week exhibited a higher prevalence of pancreatic cancer (*p* = 0.007). Furthermore, individuals who engaged in moderate exercise for 30 min on 1, 2, or 6 days per week were more common in the nonpancreatic cancer group. Meanwhile, those who exercised 3–5 days or 7 days per week exhibited a higher prevalence of pancreatic cancer (*p* = 0.044).

Charlson comorbidity index (CCI) analysis revealed significantly higher comorbidity counts among pancreatic cancer patients, except for dementia, paraplegia, severe liver disease, and human immunodeficiency virus (*p* < 0.0001). The pancreatic cancer group had a notably greater proportion of individuals with CCI scores of three or higher compared to the nonpancreatic cancer group (1621 ± 45.86 vs. 930 ± 26.31, *p* < 0.0001) (Table 1).

### 3.2 Key Factors Contributing to Pancreatic Carcinogenesis Based on Gender

This study identified 20 key variables associated with pancreatic carcinogenesis based on gender (Table 2). The results revealed noticeable gender differences. In particular, among women, significant variations were observed in height, weight, and SCr levels (*p* < 0.01). Vigorous physical activity also demonstrated notable differences among females (*p* < 0.01). In particular, women who engaged in 20 min of high‐intensity exercise once (130 ± 7.35 vs. 145 ± 8.18), twice (85 ± 4.81 vs. 116 ± 6.55), or six times per week (12 ± 0.68 vs. 16 ± 0.90) were more common in the nonpancreatic cancer group. In contrast, women who engaged in high‐intensity exercise three (95 ± 5.37 vs. 89 ± 5.02), four (44 ± 2.49 vs. 29 ± 1.64), or five times per week (63 ± 3.56 vs. 32 ± 1.81) were more prevalent in the pancreatic cancer group.

**FIGURE 2:**
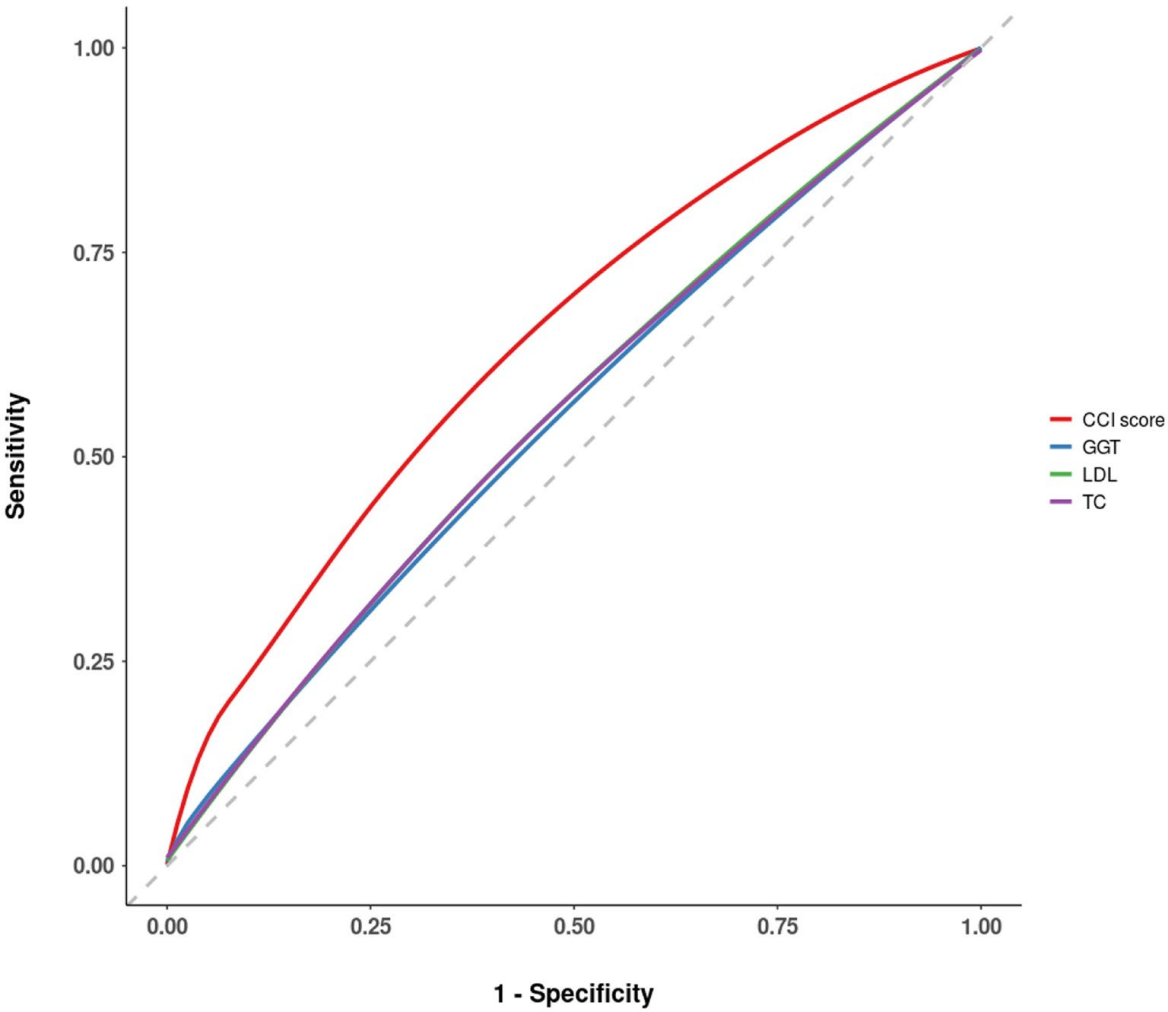
ROC curve analysis of the GGT and CCI score results. For the ROC curve analysis, variables with an AUC of 0.5 or greater, along with sensitivity and specificity greater than 0.5, were selected. The optimal threshold values of TC (AUC, 0.554), GGT (AUC, 0.546), LDL (AUC, 0.555), and CCI score (AUC, 0.637) were determined to be 179.50 (mg/dL), 29.50 (U/L), 104.50 (mg/dL), and 2.50, respectively. AUC, area under the curve; CCI, Charlson comorbidity index; GGT, gamma‐glutamyl transferase; LDL, low‐density lipoprotein; PSM, propensity score matching; ROC, Receiver operating characteristic; TC, total cholesterol.

**TABLE 2:** Gender‐specific variables prevalent in pancreatic carcinogenesis.

Variable	Male					Female				
	Pancreatic cancer		Nonpancreatic cancer		*p*	Pancreatic cancer		Nonpancreatic cancer		*p*
	*N* or mean	% or SD	*N* or mean	% or SD		*N* or mean	% or SD	*N* or mean	% or SD	
Ht	167.40	6.48	166.99	6.21	0.057	153.99	6.34	153.26	6.40	0.001
Wt	67.33	11.08	67.28	10.04	0.892	57.22	9.59	56.32	8.49	0.003
WC	84.82	8.36	84.93	7.80	0.697	80.24	9.38	79.72	8.63	0.086
BMI	23.96	3.22	24.07	2.97	0.276	24.11	3.63	23.96	3.26	0.208
SBP	127.01	14.73	127.47	14.69	0.360	125.24	16.11	125.21	16.63	0.958
DBP	77.78	9.99	78.30	9.85	0.122	76.09	9.72	76.12	10.00	0.931
Hb	14.53	1.56	14.56	1.42	0.570	12.90	1.24	12.82	1.19	0.038
FBS	115.07	41.40	104.33	26.56	< 0.0001	108.18	34.78	101.24	27.20	< 0.0001
TC	184.12	38.90	192.34	38.34	< 0.0001	195.08	41.29	201.15	39.33	< 0.0001
TG	148.54	135.45	145.81	104.55	0.502	125.42	79.03	126.11	70.72	0.782
HDL	51.98	21.48	53.24	27.09	0.125	56.23	15.64	57.00	22.50	0.239
LDL	104.29	35.22	113.69	61.45	< 0.0001	113.89	37.24	120.11	43.42	< 0.0001
SCr	1.07	0.88	1.12	1.06	0.096	0.79	0.39	0.83	0.54	0.009
SGOT	31.60	29.11	28.56	19.26	0.000	27.71	25.65	24.79	11.09	< 0.0001
SGPT	30.53	30.14	27.22	19.12	< 0.0001	24.63	28.45	21.08	13.28	< 0.0001
GGT	65.86	110.87	50.09	67.81	< 0.0001	34.95	72.54	24.04	25.96	< 0.0001
*Urine protein*										
Negative (−)	1608	91.00	1626	92.23	0.383	1645	93.04	1685	95.09	0.213
Weakly positive (±)	64	3.62	56	3.18		54	3.05	40	2.26	
Positive (+1)	49	2.77	51	2.89		43	2.43	30	1.69	
Positive (+2)	33	1.87	18	1.02		19	1.07	11	0.62	
Positive (+3)	10	0.57	10	0.57		6	0.34	5	0.28	
Positive (+4)	3	0.17	2	0.11		1	0.06	1	0.06	
*Smoking status*										
Never	568	32.14	669	37.95	< 0.0001	1681	95.08	1695	95.65	0.061
Former	611	34.58	613	34.77		19	1.07	29	1.64	
Current	588	33.28	481	27.28		68	3.85	48	2.71	
*Alcohol consumption (1 week)*										
Average	1.55	1.97	1.45	1.89	0.119	0.29	0.92	0.26	0.82	0.305
0	787	44.54	808	45.83	0.089	1504	85.07	1504	84.88	0.276
1	312	17.66	319	18.09		151	8.54	165	9.31	
2	213	12.05	243	13.78		47	2.66	60	3.39	
3	210	11.88	168	9.53		36	2.04	23	1.30	
4	59	3.34	57	3.23		10	0.57	4	0.23	
5	68	3.85	58	3.29		4	0.23	5	0.28	
6	30	1.70	42	2.38		4	0.23	3	0.17	
7	88	4.98	68	3.86		12	0.68	8	0.45	
*High‐intensity exercise (1 week)*										
Average	1.18	1.86	1.26	1.92	0.738	0.81	1.66	0.74	1.57	0.072
0	1058	59.88	1000	56.72	0.223	1298	73.42	1303	73.53	0.007
1	197	11.15	223	12.65		130	7.35	145	8.18	
2	163	9.22	188	10.66		85	4.81	116	6.55	
3	143	8.09	129	7.32		95	5.37	89	5.02	
4	49	2.77	59	3.35		44	2.49	29	1.64	
5	66	3.74	55	3.12		63	3.56	32	1.81	
6	35	1.98	39	2.21		12	0.68	16	0.90	
7	56	3.17	70	3.97		41	2.32	42	2.37	
*Moderate‐intensity exercise (1 week)*										
Average	1.48	2.06	1.51	2.07	0.194	1.23	2.01	1.11	1.90	0.182
0	947	53.59	913	51.79	0.424	1117	63.18	1138	64.22	0.145
1	183	10.36	207	11.74		147	8.31	171	9.65	
2	177	10.02	197	11.17		112	6.33	134	7.56	
3	172	9.73	166	9.42		143	8.09	122	6.88	
4	82	4.64	77	4.37		66	3.73	49	2.77	
5	80	4.53	61	3.46		75	4.24	58	3.27	
6	42	2.38	50	2.84		26	1.47	27	1.52	
7	84	4.75	92	5.22		82	4.64	73	4.12	
*CCI comorbidities*										
Acute myocardial infarction	38	2.15	39	2.21	0.900	44	2.49	18	1.02	0.001
Congestive heart failure	85	4.81	65	3.69	0.098	112	6.33	62	3.50	< 0.0001
Peripheral vascular accident	256	14.49	218	12.37	0.064	334	18.89	303	17.10	0.165
Cerebral vascular accident	259	14.66	230	13.05	0.166	298	16.86	249	14.05	0.021
Dementia	0	0.00	0	0.00	—	0	0.00	0	0.00	—
Pulmonary disease	801	45.33	632	35.85	< 0.0001	904	51.13	724	40.86	< 0.0001
Connective tissue disorder	93	5.26	51	2.89	0.000	178	10.07	109	6.15	< 0.0001
Peptic ulcer	826	46.75	617	35.00	< 0.0001	911	51.53	708	39.95	< 0.0001
Liver disease	231	13.07	98	5.56	< 0.0001	157	8.88	54	3.05	< 0.0001
Diabetes	746	42.22	407	23.09	< 0.0001	695	39.31	373	21.05	< 0.0001
Diabetes complications	231	13.07	107	6.07	< 0.0001	207	11.71	99	5.59	< 0.0001
Paraplegia	10	0.57	18	1.02	0.128	15	0.85	13	0.73	0.700
Renal disease	110	6.23	47	2.67	< 0.0001	51	2.88	38	2.14	0.160
Cancer	351	19.86	126	7.15	< 0.0001	248	14.03	137	7.73	< 0.0001
Metastatic cancer	62	3.51	15	0.85	< 0.0001	47	2.66	9	0.51	< 0.0001
Severe liver disease	16	0.91	10	0.57	0.240	14	0.79	14	0.79	0.995
HIV	0	0.00	0	0.00	—	0	0.00	0	0.00	—
CCI score	2.81	2.31	1.72	1.76	< 0.0001	2.75	2.21	1.83	1.72	< 0.0001
*CCI category*										
CCI score = 0	238	13.47	485	27.51	< 0.0001	221	12.50	439	24.77	< 0.0001
1 ≤ CCI score < 3	716	40.52	846	47.99		739	41.80	835	47.12	
3 ≤ CCI score	813	46.01	432	24.50		808	45.70	498	28.10	

*Note:* Data are presented as numbers, means, percentages, or standard deviations (SD).

Abbreviations: BMI, body mass index (kg/m^2^); CCI, Charlson comorbidity index; DBP, diastolic blood pressure (mmHg); FBS, fasting blood sugar (mg/dL); GGT, gamma‐glutamyl transferase (U/L); Hb, hemoglobin (g/dL); HDL, high‐density lipoprotein cholesterol (mg/dL); Ht, height (cm); LDL, low‐density lipoprotein cholesterol (mg/dL); NHISS DB, National Health Insurance Sharing Service database; SBP, systolic blood pressure (mmHg); SCr, serum creatinine (mg/dL); SGOT, serum glutamic oxaloacetic transaminase/aspartate aminotransferase (U/L); SGPT, serum glutamic pyruvic transaminase/alanine aminotransaminase (U/L); TC, total cholesterol (mg/dL); TG, glycerides (mg/dL); urine protein, protein in urine; WC, waist circumference (cm); Wt, weight (kg).

In terms of comorbidities, women with acute myocardial infarction, congestive heart failure, or cerebrovascular accidents exhibited a higher likelihood of pancreatic cancer (*p* < 0.05). Meanwhile, among men, smoking status was significantly associated with pancreatic cancer (*p* < 0.0001). Specifically, men who had never smoked (568 ± 32.14 vs. 669 ± 37.95) or were former smokers (611 ± 34.58 vs. 613 ± 34.77) were more prevalent in the nonpancreatic cancer group. In contrast, current smokers were more common in the pancreatic cancer group, with approximately 22% higher prevalence (588 ± 33.28 vs. 481 ± 27.28).

Variables shared between both genders included FBS, TC, LDL, SGOT, SGPT, and GGT. Furthermore, comorbidities such as pulmonary disease, connective tissue disorders, peptic ulcers, liver disease, diabetes and its complications, cancer, and metastatic cancer were more prevalent in the pancreatic cancer group. Notably, individuals with three or more of the above comorbidities were almost twice as prevalent in the pancreatic cancer group than in the nonpancreatic cancer group (*p* < 0.0001).

### 3.3 Key Risk Factors Influencing Pancreatic Carcinogenesis

Logistic regression analysis was employed to identify the primary factors associated with pancreatic carcinogenesis. Smoking status, SBP, FBS, SGPT, GGT, SCr, and the CCI score (*p* < 0.05) demonstrated significant associations with pancreatic cancer risk.

Interestingly, higher levels of SBP and SCr were associated with a reduced likelihood of pancreatic cancer, demonstrating almost 0.5% and 13.8% reductions in the risk, respectively (*p* < 0.001). Meanwhile, former smoking status was identified as a risk factor, increasing the likelihood of pancreatic cancer by approximately 24% (OR = 1.24, 95% CI = 1.073–1.437, *p* = 0.004). Furthermore, elevated FBS (OR = 1.005, *p* < 0.0001), SGPT (OR = 1.004, *p* < 0.05), GGT (OR = 1.002, *p* < 0.001), and higher CCI scores increased the risk of pancreatic cancer by 28% (*p* < 0.0001).

With respect to exercise, engaging in 20 min of high‐intensity exercise twice a week was associated with an approximate 20% reduction in pancreatic cancer risk (OR = 0.807, 95% CI = 0.656–0.993, *p* = 0.043). However, moderate‐intensity exercise did not exhibit a statistically significant association with pancreatic cancer risk.

### 3.4 Optimal Cutoff Threshold Points for Pancreatic Cancer Risk Factors Identified by ROC Curve Analysis

Following the identification of significant factors through logistic regression analysis (Tables 3 and 4), an ROC curve analysis was conducted to determine optimal thresholds for PDAC symptoms (Table 5). Out of the 21 variables under investigation, those with an area under the curve (AUC) score exceeding 0.5, along with sensitivity and specificity values above 0.4, were selected. These variables included TC, GGT, LDL, and the CCI score (Figure 2) (Table 5).

**TABLE 3:** Analysis of risk factors in pancreatic carcinogenesis.

Variable	Unadjusted				Adjusted			
	OR	95% CI		*p*	OR	95% CI		*p*
*Smoking status*								
Never	1.031	0.911	1.168	0.625	0.904	0.787	1.038	0.151
Former	1.303	1.147	1.482	< 0.0001	1.242	1.073	1.437	0.004
Current	(Ref)	(Ref)						
WC	1.003	0.997	1.008	0.314	—			
BMI	1.002	0.987	1.016	0.834	0.993	0.977	1.009	0.397
SBP	0.999	0.996	1.002	0.573	0.995	0.992	0.998	0.002
DBP	0.997	0.993	1.002	0.253	—			
*Urine protein*								
Negative (−)	0.737	0.165	3.295	0.690	1.293	0.271	6.158	0.747
Weakly positive (±)	0.922	0.201	4.219	0.917	1.389	0.285	6.780	0.685
Positive (+1)	0.852	0.185	3.920	0.837	1.104	0.225	5.419	0.903
Positive (+2)	1.345	0.281	6.427	0.711	1.546	0.302	7.914	0.601
Positive (+3)	0.800	0.153	4.184	0.792	0.820	0.145	4.650	0.823
Positive (+4)	(Ref)				(Ref)			
Hb	1.012	0.983	1.042	0.428	1.031	0.996	1.068	0.083
FBS	1.009	1.007	1.011	< 0.0001	1.005	1.004	1.007	< 0.0001
TC	0.995	0.994	0.997	< 0.0001	0.998	0.996	1.001	0.187
SGOT	1.008	1.005	1.011	< 0.0001	0.999	0.995	1.003	0.783
SGPT	1.007	1.005	1.010	< 0.0001	1.004	1.000	1.008	0.042
GGT	1.003	1.002	1.004	< 0.0001	1.002	1.001	1.002	0.001
TG	1.000	1.000	1.001	0.667	1.000	0.999	1.000	0.235
HDL	0.998	0.996	1.000	0.059	1.001	0.998	1.004	0.428
LDL	0.995	0.994	0.996	< 0.0001	0.998	0.996	1.001	0.133
SCr	0.921	0.865	0.982	0.011	0.862	0.798	0.930	0.000
CCI score	1.292	1.259	1.325	< 0.0001	1.282	1.247	1.317	< 0.0001

*Note:* Data are presented as numbers, means, percentages, or standard deviations (SD).

Abbreviations: BMI, body mass index (kg/m^2^); CCI, Charlson comorbidity index; DBP, diastolic blood pressure (mmHg); FBS, fasting blood sugar level (mg/dL); GGT, gamma‐glutamyl transferase (U/L); Hb, hemoglobin level (g/dL); HDL, high‐density lipoprotein cholesterol (mg/dL); LDL, low‐density lipoprotein cholesterol (mg/dL); SBP, systolic blood pressure (mmHg); SCr, serum creatinine (mg/dL); SGOT, serum glutamic oxaloacetic transaminase/aspartate aminotransferase (U/L); SGPT, serum glutamic pyruvic transaminase/alanine aminotransaminase (U/L); TC, total cholesterol (mg/dL); TG, glycerides (mg/dL); urine protein, protein in urine; WC, waist circumference (cm).

**TABLE 4:** Logistic regression analysis of exercise modalities and their impact on pancreatic carcinogenesis.

Variable	Unadjusted				Adjusted			
	OR	95% CI		*p*	OR	95% CI		*p*
*High‐intensity exercise (1 week)*								
0	(Ref)				(Ref)			
1	0.869	0.740	1.019	0.084	0.908	0.747	1.104	0.333
2	0.797	0.668	0.952	0.012	0.807	0.656	0.993	0.043
3	1.067	0.880	1.294	0.508	0.992	0.789	1.246	0.942
4	1.033	0.768	1.390	0.830	0.920	0.660	1.284	0.625
5	1.449	1.098	1.914	0.009	1.321	0.963	1.814	0.085
6	0.835	0.564	1.238	0.370	0.870	0.550	1.377	0.553
7	0.847	0.641	1.118	0.240	0.793	0.571	1.099	0.164
*Moderate‐intensity exercise (1 week)*								
0	(Ref)				(Ref)			
1	0.868	0.739	1.018	0.081	0.925	0.761	1.123	0.432
2	0.868	0.733	1.028	0.100	0.963	0.790	1.175	0.711
3	1.087	0.916	1.290	0.340	1.117	0.910	1.370	0.291
4	1.167	0.913	1.492	0.217	1.203	0.912	1.585	0.191
5	1.294	1.011	1.656	0.040	1.169	0.881	1.550	0.279
6	0.878	0.630	1.223	0.440	0.924	0.627	1.360	0.688
7	1.000	0.799	1.251	0.998	1.101	0.846	1.434	0.474

*Note:* Variables with significant *p* values were selected via logistic regression analysis. High‐intensity exercise = 20 min of vigorous exercise; moderate‐intensity exercise = 30 min or more of moderate exercise; number of dates with exercise (0 = none; 1 = 1 day; 2 = 2 days; 3 = 3 days; 4 = 4 days; 5 = 5 days; 6 = 6 days; 7 = everyday).

Abbreviations: CI, confidence interval; OR, odds ratio.

**TABLE 5:** ROC curve analyses for patients with pancreatic cancer.

Variable	AUC	Cutoff value	Sensitivity (%)	Specificity (%)
TC	0.554	179.50	0.428	0.656
GGT	0.546	29.50	0.419	0.650
LDL	0.555	104.50	0.477	0.610
CCI Score	0.637	2.50	0.459	0.737

*Note:* Variables with significant *p* values were identified using logistic regression analysis. Optimal cutoff points were then determined through ROC curve analysis. Sex, age, region, and healthcare insurance type were adjusted using PSM (1:1).

Abbreviations: AUC, area under the curve; CCI, Charlson comorbidity index; GGT, gamma‐glutamyl transferase; PSM, propensity score matching; ROC, receiver operating characteristic.

The optimal threshold for TC was determined to be 179.50 mg/dL (AUC = 0.554, sensitivity = 0.428, specificity = 0.656). For GGT, an optimal cutoff of 29.50 U/L was established (AUC = 0.546, sensitivity = 0.419, specificity = 0.650). LDL exhibited an optimal cutoff of 104.50 mg/dL (AUC = 0.555, sensitivity = 0.477, specificity = 0.610). Meanwhile, the CCI score presented an optimal threshold of 2.50 (AUC = 0.637, sensitivity = 0.459, specificity = 0.737).

## 4 Discussion

Leveraging the medical records from the NHISS DB, this study comprehensively analyzed the primary distinctions between pancreatic and nonpancreatic cancer patients. PSM was employed to adjust the groups based on gender, age, region, and insurance status, thus enhancing the statistical reliability of the results. Consequently, significant differences were observed between the pancreatic and nonpancreatic cancer groups in terms of biochemical indicators, lifestyle factors, and comorbidities. These findings offer critical foundational data for understanding the physiological and metabolic mechanisms associated with pancreatic cancer development.

### 4.1 Biochemical Indicators and Pancreatic Cancer Incidence

In the pancreatic cancer group, FBS, SGOT, SGPT, and GGT levels were elevated compared to the nonpancreatic cancer group. This indicates increased insulin resistance and impaired liver function in pancreatic cancer patients. These findings are consistent with those of previous studies, indicating that elevated blood sugar and liver dysfunction are significant risk factors for pancreatic cancer development [21]. Elevated FBS levels, in particular, can contribute to insulin resistance, a condition that plays a pivotal role in the metabolic characteristics of pancreatic cancer patients [22]. Diabetes mellitus (DM) is both a risk factor and a potential early manifestation of PDAC. However, it is critical to distinguish between different types of diabetes in this context.

Type 2 diabetes (T2DM), which accounts for the majority of cases in the general population, has been strongly linked to PDAC due to its association with insulin resistance and chronic hyperinsulinemia. Elevated insulin levels promote pancreatic cell proliferation and may facilitate carcinogenesis. Type 1 diabetes (T1DM), while less common, has also been associated with PDAC risk, though the exact mechanisms remain less clear. Additionally, pancreatogenic diabetes (T3cDM), which arises secondary to pancreatic disease (including chronic pancreatitis and PDAC itself), is an important but often underrecognized factor. Some patients diagnosed with new‐onset diabetes, particularly those without typical T2DM risk factors, may actually have undiagnosed PDAC. Future studies should further explore the predictive value of diabetes subtypes in PDAC screening and early detection.

Furthermore, elevated levels of liver enzymes such as SGOT, SGPT, and GGT may indicate liver dysfunction, which could either suggest the potential for liver metastasis or reflect the overall metabolic impact of pancreatic cancer on liver function. Previous studies have also established a strong association between elevated liver enzyme levels (SGOT, SGPT, and GGT) and cancer progression [23]. Furthermore, the relatively low TC and LDL levels of pancreatic cancer patients may be attributable to weight and muscle loss (cachexia), a condition commonly seen in cancer patients. This reflects the altered metabolic state associated with pancreatic cancer [24].

### 4.2 Lifestyle Factors and Pancreatic Cancer

According to the findings of this study, smoking and alcohol consumption demonstrated strong associations with the incidence of pancreatic cancer. Specifically, the pancreatic cancer group had a significantly higher proportion of current smokers compared to former smokers or individuals with no smoking history. This finding aligns with that of previous research, supporting the idea that smoking can more than double the risk of developing pancreatic cancer [25]. Current smokers, in particular, demonstrated a higher risk of developing pancreatic cancer compared to individuals from the nonpancreatic cancer group, suggesting that carcinogens in tobacco may directly impact pancreatic tissues [26].

Similarly, frequent alcohol consumption was found to be associated with an increased risk of pancreatic cancer. Individuals who consumed alcohol three or more times per week were more prevalent in the pancreatic cancer group, indicating that regular alcohol consumption may elevate the risk of pancreatic cancer. This finding is consistent with previous studies reporting that excessive alcohol intake elevates oxidative stress on pancreatic cells, which is closely linked to pancreatic cancer development [27]. These results underscore the importance of managing lifestyle factors, such as reducing smoking and limiting alcohol consumption, as key interventions for preventing pancreatic cancer.

### 4.3 Exercise and Reduced Pancreatic Cancer Risk

This study revealed noteworthy findings regarding the impact of high‐intensity exercise, particularly among women. Women who engaged in high‐intensity exercise once, twice, or six times per week were more prevalent in the nonpancreatic cancer group. This observation aligns with the findings of a cohort study conducted by Park et al. [28], who analyzed the data of 220,357 individuals from the National Health Information Database. Their findings revealed that women who engaged in high‐intensity exercise six to seven times per week had an OR of 0.47 for cancer incidence (95% CI = 0.25–0.89), indicating reduced risk [28]. This suggests that high‐intensity exercise may have beneficial effects on metabolism and immune functions, particularly among women [29].

Furthermore, our study revealed a gender‐independent association between high‐intensity exercise and reduced pancreatic cancer risk. Engaging in high‐intensity exercise at least twice a week was associated with an approximately 20% reduction in pancreatic cancer risk (OR = 0.807, *p* = 0.043). The biological mechanisms underlying this protective effect of exercise on PDAC risk may involve multiple pathways. Regular physical activity is known to reduce visceral adiposity, which plays a crucial role in systemic inflammation and insulin resistance—both of which are implicated in PDAC development. Increased visceral fat is associated with higher levels of pro‐inflammatory cytokines (e.g., IL‐6, TNF‐α) and oxidative stress, creating an environment conducive to pancreatic carcinogenesis. Additionally, exercise has been shown to modulate gut microbiota, which influences metabolic and inflammatory pathways linked to cancer risk. Studies suggest that exercise can increase microbial diversity and enhance beneficial bacterial populations, thereby improving gut barrier integrity and reducing systemic inflammation, which may contribute to PDAC prevention. These results suggest that high‐intensity exercise may aid in weight management and improve insulin resistance, thereby contributing to pancreatic cancer prevention. Previous research has also demonstrated that regular high‐intensity exercise can alleviate the risk of various cancers associated with metabolic syndrome [30]. Interestingly, no statistically significant association was observed between moderate‐intensity exercise and pancreatic cancer risk. This suggests that exercise intensity and frequency may play distinct roles in pancreatic cancer prevention, with high‐intensity exercise potentially being more effective in reducing pancreatic cancer risk.

### 4.4 Comorbidities and Importance of the CCI Score

CCI analysis revealed that pancreatic cancer patients exhibited a higher prevalence of comorbid conditions, including cardiovascular disease, respiratory disease, liver disease, and diabetes, compared to the nonpancreatic cancer group (*p* < 0.0001). Particularly, in women, specific comorbidities such as acute myocardial infarction, congestive heart failure, and cerebrovascular accidents were associated with an increased likelihood of developing pancreatic cancer (*p* < 0.05). This may be attributed to the distinctive pathophysiological interactions between cardiovascular conditions and cancer development. For instance, after an acute myocardial infarction event, elevated levels of circulating inflammatory cytokines can increase inflammation, oxidative stress, and immune dysregulation, all of which may promote cancer development in vulnerable tissues, such as the pancreas [31, 32]. In women, cerebrovascular conditions, such as stroke, may lead to autonomic nervous system imbalances, which, when combined with inflammation and hormonal changes—particularly fluctuations in estrogen levels—can alter immune function and elevate the risk of pancreatic cancer [33, 34].

Furthermore, individuals with a CCI score of three or higher were found to have more than double the risk of developing pancreatic cancer compared to those in the nonpancreatic cancer group. This suggests that a worsened overall health status, as reflected by multiple comorbidities, can elevate the likelihood of pancreatic cancer [35]. The association between multiple comorbidities and pancreatic cancer risk underscores the importance of managing and controlling these conditions as part of a preventive strategy to alleviate the risk of pancreatic cancer development.

### 4.5 Statistical Analysis of Risk Factors and Clinical Implications

Our logistic regression analysis identified smoking, SBP, FBS, SGPT, GGT, SCr, and the CCI score as significant risk factors for pancreatic cancer. Notably, elevated levels of FBS, SGPT, and GGT were strongly correlated with pancreatic cancer incidence, indicating that metabolic abnormalities may play a critical role in pancreatic cancer development [36]. Interestingly, elevated SBP and SCr levels were associated with a lower risk of pancreatic cancer. Lower SCr levels, however, may be linked to weight loss and muscle wasting, both common in pancreatic cancer patients. This weight loss can lead to reduced creatine production, which affects creatinine levels, and may partially explain this association. However, further research is required to understand the mechanisms underlying these findings and their implications for pancreatic cancer risk.

ROC analysis revealed optimal cutoff values for TC, GGT, LDL, and the CCI score, which may serve as key clinical markers for the early diagnosis of pancreatic cancer and associated prevention strategies. For instance, the identified TC cutoff of 179.50 mg/dL may improve diagnostic sensitivity and specificity. These results suggest that interventions such as tailored exercise programs targeting cholesterol management could play a preventive role in reducing pancreatic cancer risk.

As a study limitation, this study did not include chronic pancreatitis or diabetes status as variables due to data availability constraints within the NHISS DB. While both factors are well‐documented contributors to PDAC, the current dataset did not provide specific diagnostic information regarding pancreatitis history or diabetes subtypes (e.g., type 1, type 2, or pancreatogenic diabetes). As such, the impact of these conditions on PDAC risk could not be directly assessed in this analysis. Future studies integrating clinical registries or biomarker data may provide more comprehensive insights into the interplay between metabolic disorders and pancreatic carcinogenesis.

## 5 Conclusion

In summary, this study offers a comprehensive analysis of various factors associated with pancreatic cancer incidence, utilizing a large‐scale cohort from the Korean population. The study's findings highlight that lifestyle factors—such as smoking, alcohol consumption, and physical activity—can serve as major contributors to pancreatic cancer risk. The results also underscore the importance of comorbid conditions in influencing pancreatic cancer risk. Future studies should aim to elucidate the causal relationships among these risk factors across diverse population groups. The insights acquired from the current study can serve as foundational data to inform strategies for the prevention and early diagnosis of pancreatic cancer.

Given the strong associations between pancreatic cancer risk and modifiable lifestyle factors, such as smoking, alcohol consumption, and physical inactivity observed in this study, targeted interventions to promote healthier behaviors, are essential. Public health initiatives should emphasize smoking cessation programs tailored for high‐risk individuals, including structured counseling and pharmacological support. Additionally, alcohol reduction strategies, such as educational campaigns and policy interventions, can help mitigate the detrimental effects of excessive alcohol intake on pancreatic health. Encouraging regular high‐intensity exercise, as our findings suggest, could be achieved through community‐based fitness programs, mobile health applications, and physician‐led exercise prescriptions for at‐risk individuals. Furthermore, dietary interventions promoting a balanced intake of macronutrients and reduced consumption of processed foods may complement these efforts in pancreatic cancer prevention. Future research should explore the effectiveness of such targeted lifestyle modifications to validate their impact on reducing pancreatic cancer incidence and improving patient outcomes.

## Author Contributions

**Hyunseok Jee:** conceptualization, investigation, funding acquisition, writing – original draft, writing – review and editing, validation, methodology, visualization, software, formal analysis, project administration, data curation, supervision, resources.

## Consent

The author has nothing to report.

## Conflicts of Interest

The author declares no conflicts of interest.

## Supporting information


Table S1

## Data Availability

Publicly available datasets were analyzed in this study. These datasets can be found at https://nhiss.nhis.or.kr/bd/ab/bdaba000eng.do.
