# Supplementary material for: Lifestyle‐Related Risk Factors for Pancreatic Ductal Adenocarcinoma: A Longitudinal Analysis of 1,120,377 Individuals From the NHISS Cohort
Source: Cancer Med. 2025 Apr 6;14(7):e70848. doi: 10.1002/cam4.70848 (PMC11973132; doi:10.1002/cam4.70848)
Supplement: Supplementary file 1 — Table S1 [file CAM4-14-e70848-s001.docx]

**Supporting**

**Table S1. Pancreatic cancer codes used in the NHISS DB-originated analysis**

|  | Code | Classification |
| --- | --- | --- |
| Pancreatic cancer | C25 | Malignant neoplasm of pancreas |
|  | C25.0 | Malignant neoplasm of head of pancreas |
|  | C25.1 | Malignant neoplasm of body of pancreas |
|  | C25.2 | Malignant neoplasm of tail of pancreas |
|  | C25.3 | Malignant neoplasm of pancreatic duct |
|  | C25.4 | Malignant neoplasm of endocrine pancreas |
|  | C25.7 | Malignant neoplasm of other parts of pancreas |
|  | C25.8 | Malignant neoplasm of overlapping lesion of pancreas |
|  | C25.9 | Malignant neoplasm of pancreas, unspecified |
| Control | J00 | Acute nasopharyngitis |

The above pancreatic cancer-related codes (found in the Korean Standard Classification of Disease and Cause of Death, http://kssc.kostat.go.kr/ksscNew_web/index.jsp) were used for analysis in this study. The control code used in this study was J00, which represents the common cold.

Table S2. The details on variables used in this study

| Variables | 2009~2015 | | | 2016~2019 | | |
| --- | --- | --- | --- | --- | --- | --- |
|  | **Variable name** | **Code name** | **Note** | **Variable name** | **Code name** | **Note** |
| Drinking alcohol | Weekly drinking days | Q_DRK_FRQ_V09N | 0: none, 1: once, 2:  2 days, 3: 3 days | Number of times you drank alcohol in the past year | Q_DRK_PER | 1: per week |
|  |  |  |  |  |  | 2: per month |
|  |  |  | 4: 4 days, 5: 5 days, 6: 6 days, 7: everyday |  |  | 3: per year |
|  |  |  |  |  |  | 4: not drinking |
|  |  |  |  | How many times a week, a month, a year? | Q_DRK_FRQ | Number of times |
| Smoking | Smoking status | Q_SMK_YN | 1: Does not smoke | Smoking status | Q_SMK_YN | 1: Does not smoke |
|  |  |  | 2: used to smoke but quit now |  |  | 2: used to smoke but quit now |
|  |  |  | 3: still smoke now |  |  | 3: still smoke now |
| Height | Height | G1E_HGHT | cm (Missing values ​​less than 100) | Height | G1E_HGHT | cm (Missing values ​​less than 100) |
| Weight | Weight | G1E_WGHT | kg (Missing values ​​less than 20) | Weight | G1E_WGHT | kg (Missing values ​​less than 20) |
| Waist circumference | Waist circumference | G1E_WSTC | cm | Waist circumference | G1E_WSTC | cm |
| Body mass index | Body mass index | G1E_BMI | Weight (kg) / (height * height) m | Body mass index | G1E_BMI | Weight (kg) / (height * height) m |
| Systolic blood pressure | Systolic blood pressure | G1E_BP_SYS | mmHg | Systolic blood pressure | G1E_BP_SYS | mmHg |
| Diastolic blood pressure | Diastolic blood pressure | G1E_BP_DIA |  | Diastolic blood pressure | G1E_BP_DIA |  |
| Urinary protein | Urinary protein | G1E_URN_PROT | 1: Negative (－) 2: Slightly positive (±) | Urinary protein | G1E_URN_PROT | 1: Negative (－)  2: Slightly positive (±) |
|  |  |  | 3: Positive (+1) 4: Positive (+2) |  |  | 3: Positive (+1)  4: Positive (+2) |
|  |  |  | 5: Positive (＋3) 6: Positive (＋4) |  |  | 5: Positive (＋3)  6: Positive (＋4) |
| Hemoglobin | Hemoglobin | G1E_HGB | g/dL (*‘0’ value missing processing) | Hemoglobin | G1E_HGB | g/dL(*‘0’ value missing processing) |
| Preprandial blood sugar (fasting blood sugar) | Preprandial blood sugar (fasting blood sugar) | G1E_FBS | mg/dL | Preprandial blood sugar (fasting blood sugar) | G1E_FBS | mg/dL |
| Total cholesterol | Total cholesterol | G1E_TOT_CHOL | * ‘0’ value missing processing | Total cholesterol | G1E_TOT_CHOL | * ‘0’ value missing processing |
| Triglycerides | Triglycerides | G1E_TG |  | Triglycerides | G1E_TG |  |
| HDL cholesterol | HDL cholesterol | G1E_HDL |  | HDL cholesterol | G1E_HDL |  |
| LDL cholesterol | LDL cholesterol | G1E_LDL |  | LDL cholesterol | G1E_LDL |  |
| Serum creatinine | Serum creatinine | G1E_CRTN |  | Serum creatinine | G1E_CRTN |  |
| (serum GOT) AST | (serum GOT) AST | G1E_SGOT | U/L | (serum GOT) AST | G1E_SGOT | U/L |
| (serum GPT) ALT | (serum GPT) ALT | G1E_SGPT | * ‘0’ value missing processing | (serum GPT) ALT | G1E_SGPT | * ‘0’ value missing processing |
| gamma GTP | gamma GTP | G1E_GGT |  | gamma GTP | G1E_GGT |  |
| exercise | 1 week_20 minutes or more of vigorous exercise | Q_PA_VD | 0: none, 1: 1 day, 2: 2 days, 3: 3 days | 1 week_high-intensity physical activity_day | Q_PA_VD_FRQ | Day |
|  | 1 week_30 minutes or more of moderate exercise | Q_PA_MD | 4: 4 days, 5: 5 days, 6: 6 days, 7: everyday | 1 week_moderate intensity physical activity days | Q_PA_MD_FRQ | Day |

Table S3. Propensity score matching test

| Variable | Before PSM | | | | | | After PSM | | | | | |
| --- | --- | --- | --- | --- | --- | --- | --- | --- | --- | --- | --- | --- |
|  | **Pancreatic cancer** | | **Non pancratic cancer** | | **p-value** | **\|SMD\|** | **Pancreatic cancer** | | **Non pancreatic cancer** | | **p-value** | **\|SMD\|** |
|  | **(n = 3,535)** | | **(n = 226,964)** | |  |  | **(n = 3,535)** | | **(n = 3,535)** | |  |  |
|  | **N or mean** | **% or std** | **N or mean** | **% or std** |  |  | **N or mean** | **% or std** | **N or mean** | **% or std** |  |  |
| Age | 63.68 | 12.67 | 50.40 | 14.56 | <.0001 | 0.973 | 63.68 | 12.67 | 63.69 | 12.59 | 0.991 | 0.001 |
| Sex | | | | | | | | | | | | |
| - Male | 1767 | 49.99 | 108045 | 47.60 | 0.005 | 0.048 | 1767 | 49.99 | 1763 | 49.87 | 0.924 | 0.002 |
| -Female | 1768 | 50.01 | 118919 | 52.40 |  | 0.048 | 1768 | 50.01 | 1772 | 50.13 |  | 0.002 |
| Region | | | | | | | | | | | | |
| -Seoul | 455 | 12.87 | 42542 | 18.74 | <.0001 | 0.161 | 455 | 12.87 | 454 | 12.84 | 1.000 | 0.001 |
| -Pusan | 307 | 8.68 | 16265 | 7.17 |  | 0.056 | 307 | 8.68 | 307 | 8.68 |  | 0.000 |
| -Daegu | 124 | 3.51 | 11148 | 4.91 |  | 0.070 | 124 | 3.51 | 124 | 3.51 |  | 0.000 |
| -Incheon | 105 | 2.97 | 12401 | 5.46 |  | 0.124 | 105 | 2.97 | 105 | 2.97 |  | 0.000 |
| -Gwangju | 96 | 2.72 | 6392 | 2.82 |  | 0.006 | 96 | 2.72 | 92 | 2.60 |  | 0.007 |
| -Daejeon | 112 | 3.17 | 6801 | 3.00 |  | 0.010 | 112 | 3.17 | 118 | 3.34 |  | 0.010 |
| -Ulsan | 308 | 8.71 | 5329 | 2.35 |  | 0.281 | 308 | 8.71 | 308 | 8.71 |  | 0.000 |
| -Sejong | 10 | 0.28 | 509 | 0.22 |  | 0.012 | 10 | 0.28 | 5 | 0.14 |  | 0.031 |
| -Gyeonggi | 561 | 15.87 | 52615 | 23.18 |  | 0.185 | 561 | 15.87 | 564 | 15.95 |  | 0.002 |
| -Gangwon | 279 | 7.89 | 6721 | 2.96 |  | 0.219 | 279 | 7.89 | 285 | 8.06 |  | 0.006 |
| -Chungcheong-do | 106 | 3.00 | 7724 | 3.40 |  | 0.023 | 106 | 3.00 | 108 | 3.06 |  | 0.004 |
| -Chungcheongnam-do | 154 | 4.36 | 10590 | 4.67 |  | 0.015 | 154 | 4.36 | 153 | 4.33 |  | 0.001 |
| -Cheollabuk-do | 139 | 3.93 | 8891 | 3.92 |  | 0.001 | 139 | 3.93 | 136 | 3.85 |  | 0.004 |
| -Cheollanam-do | 290 | 8.20 | 8936 | 3.94 |  | 0.179 | 290 | 8.20 | 296 | 8.37 |  | 0.006 |
| -Gyeongsangbuk-do | 177 | 5.01 | 12810 | 5.64 |  | 0.028 | 177 | 5.01 | 172 | 4.87 |  | 0.006 |
| -Gyeongsangnam-do | 221 | 6.25 | 15084 | 6.65 |  | 0.016 | 221 | 6.25 | 223 | 6.31 |  | 0.002 |
| -Jeju-do | 91 | 2.57 | 2206 | 0.97 |  | 0.122 | 91 | 2.57 | 85 | 2.40 |  | 0.011 |
| Income | | | | | | | | | | | | |
| -Medicaid & NHI self-  employed/employee  subscriber low | 600 | 16.97 | 34955 | 15.40 | <.0001 | 0.043 | 600 | 16.97 | 603 | 17.06 | 0.974 | 0.002 |
| -NHI self - employed  subscriber Medium | 421 | 11.91 | 24187 | 10.66 |  | 0.040 | 421 | 11.91 | 412 | 11.65 |  | 0.008 |
| -NHI self-employed  subscriber | 477 | 13.49 | 24411 | 10.76 |  | 0.084 | 477 | 13.49 | 464 | 13.13 |  | 0.011 |
| -NHI employee  subscriber Medium | 902 | 25.52 | 78844 | 34.74 |  | 0.202 | 902 | 25.52 | 899 | 25.43 |  | 0.002 |
| -NHI employee  subscriber High | 1135 | 32.11 | 64567 | 28.45 |  | 0.080 | 1135 | 32.11 | 1157 | 32.73 |  | 0.013 |

NHI, National Health Insurance; SMD, Standardized Mean Difference; If |SMD| is less than 0.1, it is considered balanced.
